# Associations of dietary patterns and obesity with type 2 diabetes in elderly Chinese men: a machine learning approach

**DOI:** 10.3389/fnut.2025.1705683

**Published:** 2025-11-26

**Authors:** Haowei Sun, Lijin Zhu, Peng Wang, Keqing Yuan, Saida Salima Nawrin, Yufei Cui, Longfei Li

**Affiliations:** 1 College of Physical Education and Health, Heze University, Heze, Shandong, China; 2 Graduate School of Medicine, Tohoku University, Sendai, Japan

**Keywords:** type 2 diabetes mellitus, dietary patterns, obesity, unsupervised machine learning, SHAP analysis

## Abstract

**Background:**

Type 2 diabetes mellitus (T2DM) is a major global public health issue, with a particularly high prevalence in China, especially among older men. Obesity, dietary habits, and metabolic risk factors are key contributors to the development of T2DM. However, research on the relationship between dietary patterns, obesity, and T2DM in elderly Chinese men remains limited. Objective: This study aims to examine the links between obesity, dietary habits, blood pressure, and the risk of developing T2DM in elderly Chinese men. We utilize unsupervised machine learning methods along with SHAP-based model interpretation to identify significant lifestyle and metabolic factors associated with T2DM risk.

**Methods:**

A cross-sectional study was conducted with 982 participants aged 60 years and older from community health centers in Heze City, China. Unsupervised machine learning methods (UMAP) were used to identify dietary patterns, and supervised machine learning with SHAP was applied to evaluate the importance of obesity, dietary patterns, and lifestyle factors on T2DM risk. Logistic regression analyses were performed to investigate the associations between obesity, dietary habits, blood pressure, and T2DM risk. Sensitivity analyses were performed to verify the robustness of the findings.

**Results:**

Four distinct dietary patterns were identified: “high-fiber nutrient-dense,” “staple–protein,” “seafood-eggs,” and “sugary and processed foods.” The prevalence of T2DM in males was 52%. Obesity was inversely associated with T2DM odds across all models (odds ratios: 0.268–0.278, all *P* < 0.05). Compared with the high-fiber nutrient-dense pattern, adherence to the staple–protein, seafood–eggs, and sugary and processed foods patterns was significantly associated with increased obesity and T2DM risk (all *P* < 0.01). Shapley additive explanations (SHAP) analysis highlighted, physical activity level and dietary patterns as major contributors to T2DM prediction. Sensitivity analyses confirmed the robustness of these associations, independent of total caloric intake and BMI.

**Conclusion:**

In this population of elderly Chinese males, unhealthy dietary patterns are positively associated with obesity and T2DM risk, whereas obesity itself showed an inverse relationship with T2DM. These findings underscore the importance of promoting nutrient-dense diets and targeted lifestyle interventions to reduce T2DM risk in this population.

## 1 Introduction

Type 2 diabetes mellitus (T2DM) has become a significant global public health concern, with its incidence increasing consistently over recent decades (1, 2). In China, the impact of T2DM is especially severe (3), largely due to the country's rapid economic growth, accelerating urbanization, and consequent shifts in lifestyle patterns (4). These societal transformations have contributed to rising rates of obesity, unhealthy eating habits, and reduced physical activity—well-established risk factors that play a critical role in the onset of T2DM (5).

The development of type 2 diabetes mellitus is influenced by a combination of genetic susceptibility and environmental factors, reflecting its multifactorial nature (6). Among these, lifestyle behaviors that are subject to modification—such as dietary habits, physical inactivity, tobacco smoking, alcohol intake, and weight management—are recognized as key contributors to both the onset and progression of the disease (7). Increasing evidence indicates that unhealthy dietary patterns and excess body weight are closely associated with elevated blood pressure and impaired glucose metabolism, particularly among older adults, especially males (1, 8).

While numerous epidemiological studies have confirmed the protective effects of a healthy diet in reducing the risk of chronic diseases, including T2DM, most of this research has focused on Western populations. Numerous large-scale population studies have demonstrated that maintaining a nutritious diet plays a crucial role in lowering the incidence of chronic illnesses (9–11). Despite this, much of the existing research examining the link between dietary habits and chronic disease has concentrated on individual food items, limiting the ability to draw conclusions about optimal dietary patterns for health promotion (12). Dietary patterns encompass the quantity, variety, balance, and combination of various foods, beverages, and nutrients consumed regularly (13–15). It is also worth emphasizing that a significant proportion of these investigations primarily involve healthy adults or younger populations, with relatively little focus on vulnerable groups such as elderly individuals with diabetes. Recent cohort studies in China indicate that dietary patterns characterized by high intakes of vegetables, fruits, whole grains, and fish are significantly associated with a decreased risk of type 2 diabetes mellitus (T2DM), whereas consumption of diets rich in red meat, refined grains, and sodium is linked to an increased risk (16, 17). Nevertheless, evidence regarding these dietary associations among elderly Chinese populations, especially males—a demographic considered particularly vulnerable and understudied—remains limited.

To address these gaps, advanced data-driven approaches such as unsupervised machine learning have been increasingly applied in nutritional epidemiology. Unlike traditional statistical methods (e.g., PCA or factor analysis), which require predefined assumptions about dietary groupings, unsupervised learning can uncover previously hidden or emergent dietary patterns in an unbiased manner, providing a more nuanced representation of real-world eating behaviors. In particular, Uniform Manifold Approximation and Projection (UMAP) has demonstrated superior capability in capturing non-linear relationships and preserving both local and global structure within high-dimensional dietary data, enabling more accurate characterization of complex dietary patterns (18–21). Moreover, Shapley Additive Explanations (SHAP) enable the interpretation of complex models by quantifying the contribution of individual features to predicted outcomes, thereby enhancing both transparency and clinical relevance (22). Despite their potential, relatively few studies have employed these methodologies to examine dietary patterns (23, 24). This unsupervised methodology facilitates the data-driven identification of naturally occurring dietary patterns, as opposed to relying on predefined categories, thereby uncovering subtle and previously unrecognized relationships between diet and disease.

Therefore, the present study aims to examine the associations between obesity, dietary patterns, blood pressure, and T2DM risk among elderly Chinese males. By leveraging UMAP for dietary pattern extraction and SHAP for model interpretability, this study seeks to identify key nutritional and metabolic predictors of T2DM and provide evidence to inform targeted prevention strategies and public health interventions in China.

## 2 Materials and methods

### 2.1 Study design and participants

This cross-sectional study was conducted to investigate the associations between obesity, dietary patterns, blood pressure, and the risk of type 2 diabetes mellitus in elderly Chinese males. A total of 982 participants aged ≥60 years were recruited from community health centers in Heze City between 2018 and 2019. Participants were selected using a multistage stratified random sampling method to ensure representativeness. Of the 982 participants, 453 (46.1%) were rural residents and 529 (53.9%) were urban residents.

Inclusion criteria were: (1) male sex, aged ≥60 years; (2) residence in the study area for at least 1 year; and (3) ability to provide informed consent. Exclusion criteria included: (1) diagnosis of type 1 diabetes; (2) severe cardiovascular, renal, or hepatic disease; (3) cognitive impairment that affected the ability to complete dietary assessments; and (4) use of medications that significantly alter metabolic function. The study was conducted in accordance with the Declaration of Helsinki, and all procedures were approved by the ethics committee of Heze University. All participants or their families signed informed consent documentation before sample collection. The study was approved by the Institutional Review Board of Heze University (Approval No. 20180314).

### 2.2 Diagnosis of obesity

Anthropometric measurements were conducted following standardized protocols. Height and weight were measured using a calibrated stadiometer and a digital scale, respectively, with participants wearing light clothing and no shoes. Body mass index (BMI) was calculated as weight (kg) divided by height squared (m^2^) (25). Subjects were categorized into four BMI groups according to the World Health Organization guidelines for Asians: underweight (< 18.5 kg/m^2^), normal weight (18.5–22.9 kg/m^2^), overweight (23–24.9 kg/m^2^), and general obesity (≥25 kg/m^2^) (26). Abdominal obesity was defined as a WC ≥90 cm in men (27). Obesity was further defined as BMI ≥28 kg/m^2^, based on the criteria recommended by the Working Group on Obesity in China (WGOC) (28). Central obesity was defined as waist circumference (WC) ≥90 cm for men (29).

### 2.3 Blood pressure

Blood pressure (BP) was measured using an automated sphygmomanometer after participants has rested for at least 5 min in a seated position. Three measurements were taken at 1-min intervals, and the average of the last two readings was recorded. Hypertension was defined as systolic BP ≥140 mmHg, diastolic BP ≥90 mmHg, or current use of antihypertensive medication (30).

### 2.4 Diagnosis of diabetes

Fasting blood samples were collected in the morning after at least 8 h of overnight fasting. Blood glucose, glycated hemoglobin (HbA1c), and lipid profiles (total cholesterol, triglycerides, high-density lipoprotein cholesterol, and low-density lipoprotein cholesterol) were analyzed using standard enzymatic methods in a certified laboratory. Type 2 diabetes mellitus was defined according to the American Diabetes Association criteria as fasting plasma glucose (FPG) ≥7.0 mmol/L, HbA1c ≥6.5%, self-reported physician diagnosis, or current use of antidiabetic medication (31).

### 2.5 Dietary assessment and pattern analysis

A validated semi-quantitative food frequency questionnaire (FFQ) comprising of 81 items was used to assess food and nutrient intake (32). Trained dietitians conducted structured interviews in which participants were asked to recall the frequency of their consumption of each food item over the previous 4 weeks (33). To facilitate accurate estimation of portion sizes, trained interviewers used food photographs depicting standard reference portions. For liquid items such as milk, juice, and other beverages, intake was recorded in milliliters (ml) and subsequently converted to grams for analysis purposes (34). The frequency of food intake was recorded using seven categories: (1) never; (2) less than once per week; (3) once per week; (4) 2–3 times per week; (5) 4–6 times per week; (6) once per day; and (7) two or more times per day. The 81 food items were classified into 21 predefined food groups according to similarities in nutrient profiles, culinary use, and grouping schemes adopted in previous studies (32, 35). The average intake of each food item was calculated by multiplying the reported frequency by the portion size, using data from the China Food Composition table (2009) as a reference (36).

### 2.6 Clustering dietary patterns using machine learning

To identify dietary patterns, an unsupervised machine-learning framework combining nonlinear dimensionality reduction, clustering, and factor analysis was applied. Dietary intake data derived from the validated FFQ were first aggregated into 21 predefined food groups and standardized prior to analysis. UMAP was not used for variance decomposition or factor extraction. Uniform manifold approximation and projection (UMAP) was employed solely as a nonlinear dimensionality-reduction and visualization tool to project the high-dimensional dietary intake data into a low-dimensional space while preserving local neighborhood structure. UMAP was not used for variance decomposition or factor extraction. The UMAP parameters were specified a priori as follows: n_neighbors = 10, min_dist = 0.1, n_components = 2, and Chebyshev distance as the similarity metric (37). These values were optimized through systematic parameter tuning to ensure the generation of distinct and well-separated clusters.

Clustering was subsequently performed using the *K*-means algorithm applied to the two-dimensional UMAP embedding. The optimal number of clusters (*K*) was initially explored using the elbow method based on within-cluster sum of squares calculated in the UMAP-reduced space. As a complementary, descriptive aid, kernel density contour maps were visually inspected to assess the distribution of observations in the two-dimensional space; however, final cluster selection was primarily guided by the elbow criterion and interpretability. *K*-means clustering was implemented using default initialization and convergence settings. To assess the robustness of dietary pattern clusters derived from *K*-means on the UMAP embedding, we generated 500 bootstrap samples of the original dataset, applied *K*-means clustering with the same number of clusters and UMAP parameters to each sample, and quantified cluster stability as the average proportion of times participants were assigned to the same cluster across all iterations. Silhouette scores were computed in the original feature space, indicating moderate cluster cohesion and separation across the derived dietary patterns.

To characterize the dietary composition underlying each cluster, exploratory factor analysis with varimax rotation was conducted on the original 21 food-group variables, rather than on the UMAP-reduced dimensions. Factor analysis was used exclusively for *post hoc* interpretation of dietary patterns and not for cluster construction. Factor loadings with absolute values ≥0.25 were considered meaningful contributors to each pattern. Dietary clusters were subsequently labeled based on the dominant food groups reflected in the factor-loading structure. Standardized factor scores were summarized descriptively across clusters to illustrate relative dietary tendencies.

To complement the unsupervised identification of dietary patterns, a supervised machine-learning approach was applied to evaluate their association with Type 2 diabetes mellitus (T2DM). Specifically, dietary cluster membership derived from the UMAP–*K*-means procedure was included as an exposure variable, together with selected demographic, anthropometric, and lifestyle covariates, in an extreme gradient boosting (XGBoost) classifier. This combined analytical strategy enabled data-driven identification of dietary profiles and exploratory assessment of their predictive relevance for T2DM.

Model performance was evaluated using five-fold cross-validation, and hyperparameters were optimized through grid search. To enhance interpretability, SHapley Additive exPlanations (SHAP) were computed to quantify the relative contribution of each predictor to the model output. SHAP summary plots and force plots were generated to provide local, individual-level explanations of model predictions, illustrating how each feature contributed to deviations from the baseline risk. Categorical variables were transformed into binary dummy variables using one-hot encoding prior to model training. Consequently, SHAP force plots reflect the contribution of category membership rather than any ordinal numerical effect.

Unsupervised machine learning algorithms were implemented using the Python programming language (version 3.7.10) within the Anaconda distribution (version 2.1.1) on a Mac operating system (macOS 12.5). The required libraries, including *umap-learn* (version 0.3.10) and *scikit-learn* (version 0.21.3), were installed and executed in a Jupyter Notebook environment (version 6.4.6).

### 2.7 Statistical analysis

Statistical analyses were conducted using JMP software (version 16.2.0) and Python (version 3.7.10). The distribution of variables was assessed for normality using the Kolmogorov–Smirnov test. Since all continuous variables met the assumption of normality, no transformations were necessary. Descriptive statistics were calculated for each cluster, with means and standard deviations reported for continuous variables, and frequencies with percentages for categorical variables. To examine differences related to T2DM, analysis of variance (ANOVA) was applied to continuous data, while Chi-square tests were utilized for categorical variables.

Other lifestyle-related and sociodemographic information—including age, smoking status, sleep quality, sleep duration, living arrangements, and educational level—was obtained through a self-administered questionnaire. Average sleep duration per night was estimated based on participants' self-reported bedtime and wake-up time, without differentiating between weekdays and weekends. Sleep duration was then categorized into two groups: “6–8 h per day” (considered typical) and “atypical” (shorter or longer than 6–8 h). Educational attainment was defined by the highest level of formal education completed and categorized into four groups: “Illiterate,” “Primary school,” “Middle school,” and “College or above.” Physical activity was assessed using the International Physical Activity Questionnaire (IPAQ). Total weekly physical activity was calculated in terms of metabolic equivalents (METs) multiplied by hours per week (METs × h/week). Blood samples were collected in siliconized vacuum tubes containing sodium fluoride for biochemical analysis. Fasting blood glucose (FBG) was measured using an enzymatic method (Eerotec). Serum uric acid (UA) levels were determined enzymatically using the Pureauto SUA kit (Sekisui Medical Co. Ltd.). Triglycerides, low-density lipoprotein cholesterol (LDL-C), and high-density lipoprotein cholesterol (HDL-C) levels were also measured using enzymatic methods with appropriate reagent kits. Participants reported any history of physical illness and current use of medication, including treatment for diabetes and dyslipidemia, via a binary (“yes” or “no”) format. A two-tailed *P*-value < 0.05 was considered statistically significant.

### 2.8 Sensitivity analyses

To evaluate the robustness of our findings, we conducted sensitivity analyses focusing on BMI and total energy intake. Participants were stratified by BMI categories (underweight, normal weight, overweight, and obese), and multivariate logistic regression models were rerun to assess whether associations between obesity and T2DM were consistent across BMI strata. Additionally, total daily energy intake was estimated from the validated food frequency questionnaire (FFQ), and participants were divided into quartiles based on energy intake. Multivariate logistic regression models were subsequently reanalyzed with total energy intake included as a covariate to determine whether the observed associations between dietary patterns, obesity, blood pressure, and T2DM risk remained robust after adjustment for caloric intake. These sensitivity analyses served as an internal validation approach to examine the robustness of the observed associations under different model assumptions, thereby increasing confidence in the stability of the findings.

## 3 Results

### 3.1 Descriptive characteristics by type 2 diabetes mellitus

As shown in Table 1, 52% of the male participants had Type 2 diabetes mellitus. Notably, an inverse association between obesity and T2DM was observed in subsequent analyses (Table 1). No statistically significant differences were observed between diabetic and non-diabetic males regarding age, body mass index (BMI), hip and waist measurements, level of physical activity, systolic and diastolic blood pressure, income, serum insulin, HOMA-IR values, LDL and HDL cholesterol, triglyceride levels, C-reactive protein (CRP), uric acid concentration, fasting plasma glucose, total caloric intake, glycated hemoglobin (HbA1c), education level, tobacco and alcohol use, family history of diabetes, duration and quality of sleep, marital and employment status, residential area, presence of general or abdominal obesity, or ethnic background. Nonetheless, males with diabetes were more frequently prescribed antidiabetic drugs and had a higher likelihood of reporting a family history of hypertension compared with those without T2DM (*P* < 0.05).

**Table 1:** Demographic characteristics of the diabetes.

Characteristics	Male		
	T2DM status	*P*-value	
	**No (471, 48%)**	**Yes (511, 52%)**	
Age	74.21 ± 8.65	74.48 ± 8.78	0.624
BMI	26.85 ± 4.75	26.67 ± 4.89	0.562
Hip circumference	104.86 ± 14.52	105.32 ± 14.51	0.625
Waist circumference	94.40 ± 14.89	95.19 ± 14.85	0.722
Physical activity	1556.02 ± 846.16	1498.43 ± 865.29	0.293
SBP	136.63 ± 26.12	133.97 ± 26.21	0.112
DBP	84.91 ± 14.71	85.29 ± 14.00	0.684
Income	5416.94 ± 2560.97	5428.83 ± 2637.67	0.594
Serum insulin	13.69 ± 6.60	13.76 ± 6.80	0.874
Homa-IR	3.27 ± 1.63	3.20 ± 1.59	0.508
HDL	60.27 ± 16.79	60.15 ± 17.11	0.908
LDL	126.00 ± 42.62	124.06 ± 43.21	0.479
Triglycerides	167.38 ± 71.75	177.67 ± 72.53	0.126
CRP	5.09 ± 2.84	5.11 ± 2.87	0.963
Uric acid	5.49 ± 1.45	5.57 ± 1.45	0.439
Total energy intake	2467.25 ± 573.65	2532.60 ± 562.92	0.072
hbA1c	5.32 ± 1.68	8.46 ± 1.45	0.001
Fasting blood glucose	136.48 ± 37.42	133.97 ± 36.70	0.039
Educational level (*N*, %)			
Illiteracy	114 (24.2%)	138 (27.0%)	0.106
Primary school	106 (22.5%)	138 (27.0%)	
Middle school	127 (27.0%)	128 (25.0%)	
College or above	124 (26.3%)	107 (20.9%)	
Smoking status (*N*, %)			
Never	172 (36.5%)	167 (32.7%)	0.248
Former	165 (35.0%)	175 (34.2%)	
Current	134 (28.5%)	169 (33.1%)	
Alcohol consumption (*N*, %)			
No	66 (14.0%)	85 (16.6%)	0.564
Drinker	405 (86.0%)	426 (83.4%)	
Use of antidiabetic medication (*N*, %)			
No	235 (49.9%)	249 (48.7%)	0.034
Yes	236 (50.1%)	262 (51.3%)	
Family history of diabetes (*N*, %)			
No	231 (49.0%)	256 (50.1%)	0.742
Yes	240 (51.0%)	255 (49.9%)	
Family history of hypertension (*N*, %)			
No	266 (56.5%)	250 (49.1%)	0.018
Yes	205 (43.5%)	261 (51.1%)	
Sleep duration (*N*, %)			
< 7 h	234 (49.7%)	261 (51.1%)	0.866
7–9 h	169 (35.7%)	174 (34.1%)	
>9 h	69 (14.6%)	76 (14.9%)	
Sleep quality (*N*, %)			
Poor	164 (34.8%)	157 (30.7%)	0.171
Good	307 (65.2%)	354 (69.3%)	
Marital status (*N*, %)			
Other	239 (50.7%)	272 (53.2%)	0.720
Married	232 (49.3%)	239 (46.8%)	
Work status (*N*, %)			
Unemployed	136 (28.9%)	163 (31.9%)	0.566
Employed	335 (71.1%)	348 (68.1%)	
Residency (*N*, %)			
Rural area	205 (51.4%)	248 (50.9%)	0.876
Urban area	229 (48.6%)	251 (49.1%)	
General obesity (*N*, %)			
No	229 (48.6%)	240 (47.0%)	0.604
Yes	242 (51.4%)	271 (53.0%)	
Central obesity (*N*, %)			
No	256 (54.4%)	253 (49.5%)	0.129
Yes	215 (45.6%)	258 (50.5%)	
Ethnicity (*N*, %)			
Minority	239 (50.7%)	252 (49.3%)	0.655
Han Chinese	232 (49.3%)	259 (50.7%)	
Cardiovascular disease (*N*, %)			
No	237 (50.3%)	255 (49.9%)	0.8963
Yes	234 (49.7%)	256 (50.1%)	

This table presents the demographic data, blood test results, and clinical characteristics related to diabetes symptoms. Group comparisons were performed using Chi-square tests or analysis of variance (ANOVA), with means and standard deviations (mean ± SD) shown in parentheses. *P* values are derived from one-way ANOVA or Chi-square tests, as appropriate. BMI, body mass index; SBP, systolic blood pressure; DBP, diastolic blood pressure; FBG, fasting blood glucose; RBC, red blood cells; HB, hemoglobin; WBC, white blood cells; PA, physical activity; LDL-C, low-density lipoprotein cholesterol; HDL-C, high-density lipoprotein cholesterol.

### 3.2 Clustering dietary patterns using unsupervised machine learning

**Figure 1:**
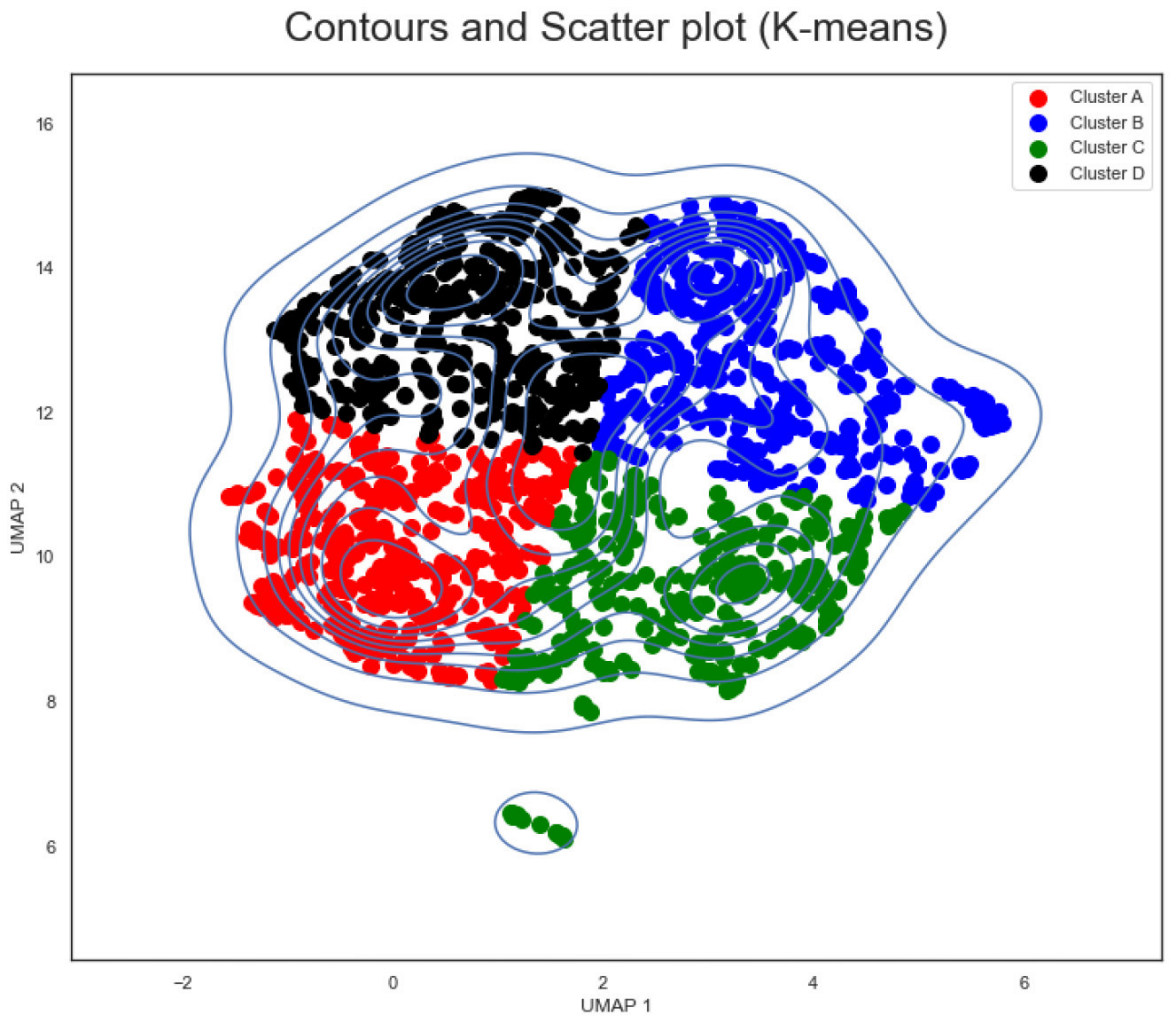
Scatter plot and contour map. The data has been reduced to two dimensions [Dimension 1 (D1) and Dimension 2 (D2)] using UMAP. Each point on the graph represents an individual participant in the study. The contour lines are curves that show areas of constant Gaussian values in the 2D space, connecting points of similar 2D values. Number clusters (*n* = 4) were chosen optimally by contour map analysis. Colors indicate cluster assignment using *K*-means clustering (*K* = 4; see Section 2 for details).

A dataset with 81 dimensions (81 variables) was reduced to two dimensions using the UMAP algorithm. The first and second dimensions were plotted on the *x* and *y* axes. A 2D scatter plot of the clusters is presented in Figure 1. A density contour map overlaid on the scatter plot revealed four distinct peaks, indicating four natural dietary clusters. These clusters represent four principal dietary patterns in the study population.

Cluster robustness was assessed using silhouette scores in the standardized original feature space, with 500 bootstrap resamples used to compute 95% confidence intervals. As shown in Supplementary Table S7, the highest mean score was observed for *K* = 4 (0.46, 95% CI: 0.43–0.49). Scores for *K* = 3 and *K* = 5 were lower (0.36 and 0.39), indicating that *K* = 4 is the optimal cluster number. Analyses across different UMAP parameter settings (n_neighbors = 10–50, min_dist = 0.1–0.5) and alternative *K* values confirmed that the clustering structure remained stable.

### 3.3 UMAP-derived visualization of four principal dietary patterns based on factor loadings

To investigate dominant dietary behavior patterns, we conducted a factor analysis combined with Uniform Manifold Approximation and Projection (UMAP), using categorized food groups as the basis for analysis. The derived factor loadings for each dietary pattern reveal key food items that contributed significantly, either positively or negatively (loadings ≥0.25 or ≤ −0.25) are shown in Table 2. The first pattern, referred to as “high-fiber nutrient-dense,” was characterized by high consumption of vegetables, legumes, mushrooms, tubers, fruits, whole grains, seaweed, wheat-based products, nuts, and dairy. The second pattern, labeled “staple–protein,” was associated with greater intake of white rice, red and poultry meats, organ meats, eggs, legumes, and various condiments. The third pattern, termed “seafood-eggs,” was marked by frequent consumption of seafood, eggs, dairy, nuts, fruits, and tea, while showing an inverse relationship with sugar-sweetened beverages. The fourth dietary pattern, described as “sugary and processed foods,” was characterized by high intake of fast food, sugary drinks, desserts, and alcoholic beverages, alongside lower consumption of dairy and white rice. Both high-fiber nutrient-dense and seafood–eggs patterns provide high-quality protein and fiber, but the former emphasizes vegetables and whole grains, while the latter focuses on seafood and eggs. The staple–protein pattern relies on refined carbohydrates and red meat, with moderate sugar intake. The sugary and processed foods pattern is energy-dense but lacks fiber and high-quality protein. Collectively, these four dietary patterns accounted for 12.33, 24.62, 33.03, and 39.10% of the total variance in dietary intake, respectively.

**Table 2:** Description of dietary patterns identified through factor analysis and UMAP.

Dietary patterns	Factor loading coefficient	Cumulative variance explained (%)
Food		
High-fiber nutrient-dense pattern		
Vegetables	0.69	12.33
Beans	0.65	
Mushrooms	0.63	
Tubers	0.58	
Fruits	0.56	
Coarse cereal	0.50	
Seaweed	0.36	
Wheat	0.40	
Nuts	0.29	
Dairy products	0.21	
Staple–protein pattern		
Red meat	0.78	24.62
White rice	0.60	
Poultry	0.59	
Animal organ meat	0.45	
Condiments	0.35	
Eggs	0.28	
Beans	0.34	
Seafood–eggs pattern		
Eggs	0.67	33.03
Seafood	0.55	
Dairy products	0.33	
Nuts	0.30	
Fruits	0.29	
Tea	0.26	
Beverages	−0.25	
Sugary and processed foods pattern		
Fast food	0.73	39.10
Beverages	0.70	
Dessert	0.61	
Alcoholic beverages	0.35	
White rice	−0.25	
Dairy products	−0.26	

### 3.4 Importance of difference features interpreted by SHAP value

**Figure 2:**
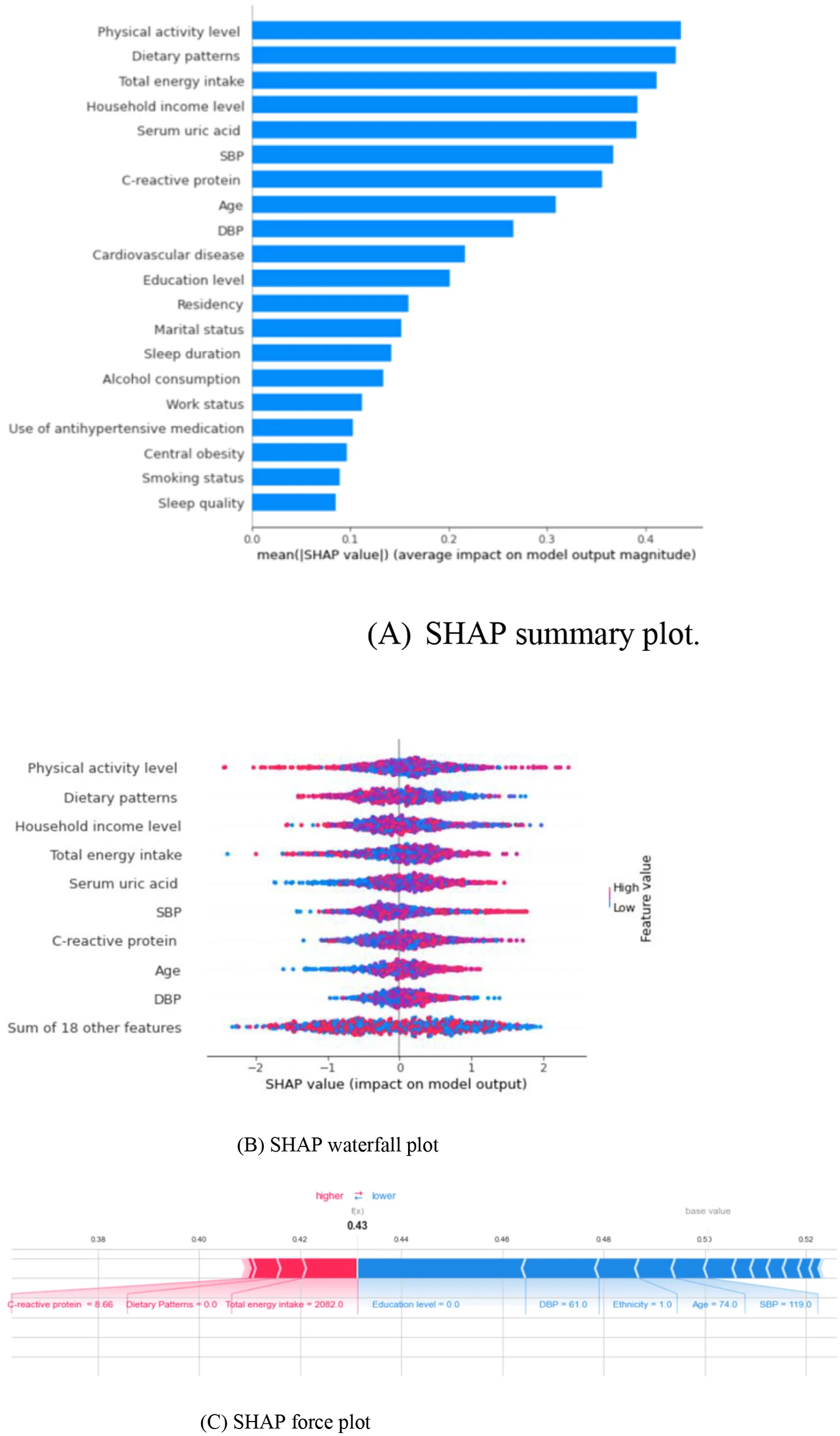
SHAP values of dietary patterns for T2DM model. **(A)** SHAP summary plot. Absolute feature contribution of the 20 features with the highest mean absolute SHAP value. **(B)** SHAP waterfall plot. Complete distribution of the SHAP values for the top 20 features based on the highest mean absolute SHAP value. Each sample of the test set is represented as a data point per feature, and the *x* axis shows the positive or negative effect on the model's prediction of the feature. The color coding depicts the value of the feature and is scaled independently based on the range observed in the data. **(C)** SHAP force plot. Representative force plots, depicting how the features contributed to the prediction for specific data points.

The SHAP visualization (Figure 2) illustrates the relative contribution of the top 20 features utilized by an XGBoost machine learning model in predicting the risk of Type 2 diabetes mellitus in elderly Chinese males. The model was trained using a comprehensive feature set, including demographic information, anthropometric measures, blood pressure, dietary intake, and physical activity variables. Its performance was evaluated via five-fold cross-validation, achieving an average ROC of 0.83 and an accuracy of 84% (see Supplementary Figure 1), indicating a high level of discriminatory capability. Notably, physical activity level and dietary patterns were identified as the primary influential factors in the model predictions, showing the most substantial contributions among the examined features. To better elucidate how dietary patterns influence the model's predictions and to clarify the underlying mechanisms, we used the shap-viz package to produce force plots (also shown in Figure 2). This figure displays the ranked influence of each feature on T2DM risk and conveys the overall predictive magnitude. In the visualization, red-colored features denote variables that contribute to a decreased probability of T2DM onset. These visual outputs enhance model interpretability and support users in understanding the basis of predictions, thereby facilitating more informed dietary choices.

### 3.5 Association of clusters with type 2 diabetes mellitus and obesity

Table 3 displays the outcomes of five sequential logistic regression models assessing the link between obesity and the risk of Type 2 diabetes mellitus (DM) based on a cross-sectional analysis. Throughout all models, obesity was consistently found to be inversely related to the risk of T2DM, as evidenced by odds ratios between 0.268 and 0.278, all with *P*-values under 0.05. This negative association remained robust even with the addition of covariates in each subsequent model.

**Table 3:** Multivariable logistic regression analysis of the association between obesity and type 2 diabetes mellitus.

Model	Obeslty	T2DM	*P*-value
Model 1	1 (ref)	0.268 (0.081, 0.458)	0.012
Model 2	1 (ref)	0.271 (0.084, 0.469)	0.014
Model 3	1 (ref)	0.275 (0.083, 0.477)	0.015
Model 4	1 (ref)	0.278 (0.084, 0.474)	0.015
Model 5	1 (ref)	0.278 (0.083, 0.475)	0.015

Model 1: crude model; Model 2: adjust for age, smoking status, marital status, work status, residency, ethnicity, household income level, alcohol consumption, physical activity, sleep duration; Model 3: adjust for blood pressure, HDL, LDL, triglycerides, CRP, serum uric acid; Model 4: adjust for cluster; Model 5: adjust for family history of hypertension, family history of diabetes, use of antihypertensive medication.

In the multivariable logistic regression analysis, dietary patterns were significantly associated with obesity odds among elderly Chinese males (Table 4). Using the high-fiber nutrient-dense dietary pattern as the reference, participants adhering to the staple–protein pattern exhibited a higher odds of obesity across all models (Model 1: β = 0.351, 95% CI: 0.119–0.585, *P* = 0.003; Model 5: β = 0.360, 95% CI: 0.118–0.569, *P* = 0.002). Similarly, the seafood–eggs pattern was positively associated with obesity (Model 1: β = 0.414, 95% CI: 0.181–0.648, *P* = 0.003; Model 5: β = 0.386, 95% CI: 0.109–0.613, *P* = 0.002). Notably, the sugary and processed foods pattern demonstrated the strongest association with obesity odds, with consistently significant effects after full adjustment (Model 1: β = 0.610, 95% CI: 0.368–0.831, *P* = 0.003; Model 5: β = 0.583, 95% CI: 0.358–0.864, *P* = 0.002). These associations remained robust after adjusting for a wide range of sociodemographic, lifestyle, biochemical, and clinical covariates, suggesting that dietary patterns characterized by lower nutritional quality are independently linked to increased obesity odds in this population.

**Table 4:** Multivariable logistic regression analysis of the association between dietary patterns and obesity.

Model	Cluster A (high-fiber nutrient-dense)	Cluster B (staple–protein)	Cluster C (seafood–eggs)	Cluster D (sugary and processed foods)	*P*-value
Model 1	1 (ref)	0.351 (0.119, 0.585)	0.414 (0.181, 0.648)	0.610 (0.368, 0.831)	0.003
Model 2	1 (ref)	0.368 (0.148, 0.589)	0.433 (0.190, 0.654)	0.616 (0.381, 0.848)	0.001
Model 3	1 (ref)	0.390 (0.166, 0.618)	0.438 (0.207, 0.669)	0.630 (0.410, 0.856)	0.001
Model 4	1 (ref)	0.362 (0.146, 0.592)	0.431 (0.169, 0.648)	0.590 (0.370, 0.824)	0.002
Model 5	1 (ref)	0.360 (0.118, 0.569)	0.386 (0.109, 0.613)	0.583 (0.358, 0.864)	0.002

Model 1: crude model; Model 2: adjust for age, smoking status, marital status, work status, residency, ethnicity, household income level, alcohol consumption, physical activity, sleep duration; Model 3: adjust for fasting blood glucose, HbA1c, serum insulin, HDL, LDL, triglycerides, CRP, serum uric acid; Model 4: adjust for family history of hypertension, family history of diabetes, use of antihypertensive medication.

As shown in Table 5, all three non-reference dietary patterns were significantly associated with increased odds of type 2 diabetes mellitus (T2DM) compared with the high-fiber nutrient-dense pattern. In Model 1, ORs were 1.32 (95% CI: 1.04–1.64) for staple–protein, 1.26 (95% CI: 1.01–1.62) for seafood–eggs, and 1.54 (95% CI: 1.21–1.97) for sugary and processed foods, with *P* = 0.012. These associations remained significant across Models 2–5 after progressively adjusting for demographic factors, lifestyle behaviors, metabolic biomarkers, blood pressure, T2DM status, and family history or medication use (all *P* < 0.01), indicating a consistent positive relationship between unhealthy dietary patterns and T2DM odds in this population.

**Table 5:** Multivariable logistic regression analysis of the association between dietary patterns and type 2 diabetes mellitus.

Model	Cluster A (high-fiber nutrient-dense)	Cluster B (staple–protein)	Cluster C (seafood–eggs)	Cluster D (sugary and processed foods)	*P*-value
Model 1	1 (ref)	1.321 (1.041, 1.644)	1.268 (1.013, 1.621)	1.546 (1.213, 1.977)	0.012
Model 2	1 (ref)	1.341 (1.072, 1.651)	1.256 (1.018, 1.559)	1.482 (1.164, 1.925)	0.006
Model 3	1 (ref)	1.376 (1.091, 1.712)	1.276 (1.046, 1.603)	1.460 (1.123, 1.862)	0.008
Model 4	1 (ref)	1.388 (1.122, 1.768)	1.317 (1.066, 1.664)	1.455 (1.118, 1.854)	0.006
Model 5	1 (ref)	1.412 (1.112, 1.765)	1.336 (1.084, 1.757)	1.494 (1.106, 1.884)	0.005

Model 1: crude model; Model 2: adjust for age, BMI, smoking status, marital status, work status, residency, ethnicity, household income level, alcohol consumption, physical activity, sleep duration; Model 3: adjust for HDL, LDL, triglycerides, CRP, serum uric acid; Model 4: adjust for blood pressure; Model 5: adjust for family history of hypertension, family history of diabetes, use of antihypertensive medication.

### 3.6 Sensitivity analysis

To assess the robustness of our findings, sensitivity analyses were performed by categorizing participants according to BMI and dividing them into quartiles based on total daily energy intake, estimated using the validated FFQ. Multivariate logistic regression models were then rerun with BMI and energy intake included as covariates. The results indicated that these adjustments had minimal impact on the associations between dietary patterns, obesity, blood pressure, and T2DM risk, with effect sizes and statistical significance largely unchanged compared with the original models (see Supplementary Tables 1–6). The results of the sensitivity analyses were consistent with the primary findings, indicating that the associations remained robust across different BMI strata and after adjustment for total energy intake.

### 3.7 Age- matched analysis

**Figure 3:**
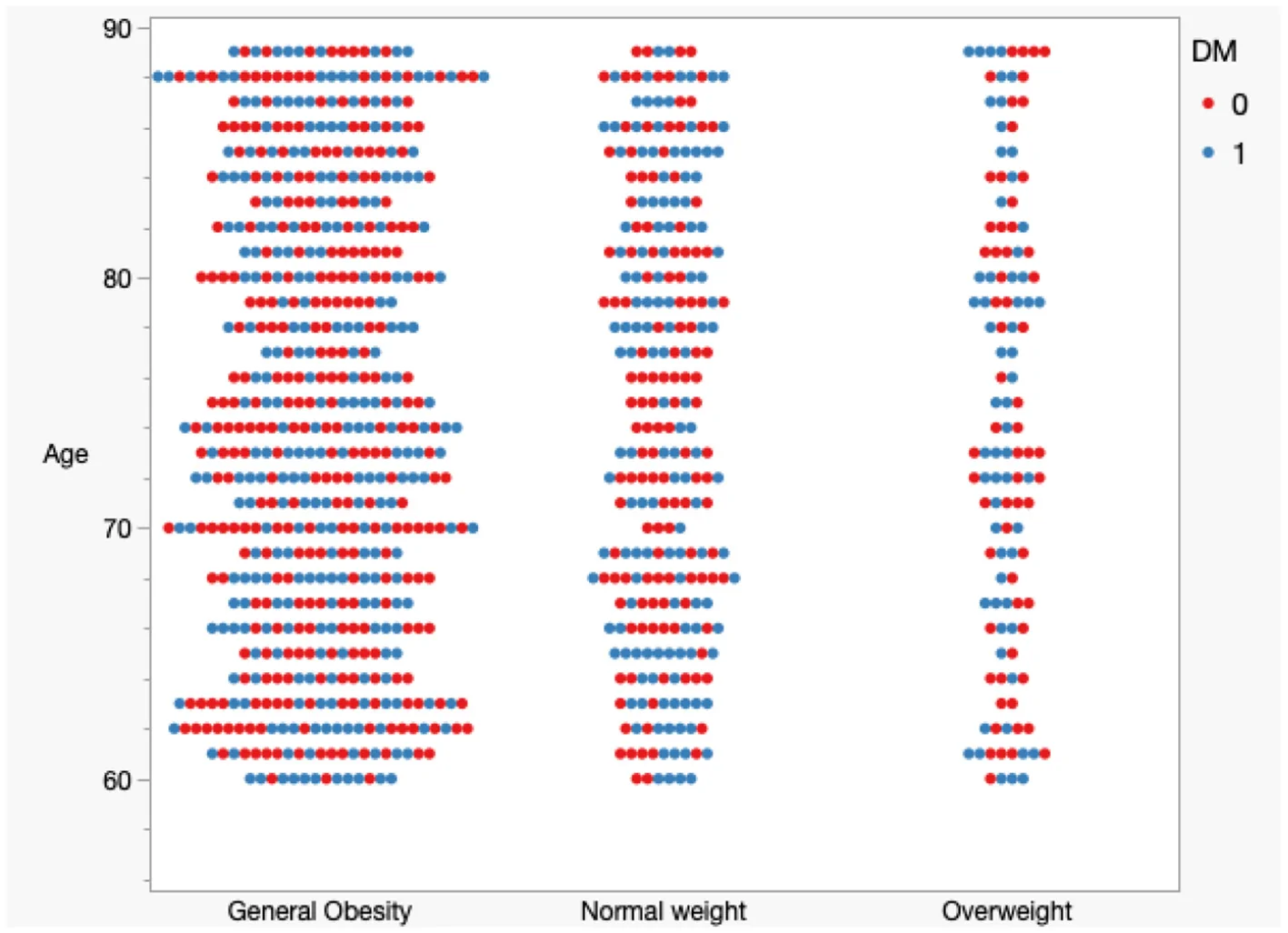
A dot plot was used to illustrate the age distribution across different scales. The color coding represents each scale distinctly.

As shown in Figure 3 (Supplementary materials), the distribution of Type 2 diabetes mellitus was imbalanced across different levels of obesity and clusters.

## 4 Discussion

This study explored the associations between obesity, dietary patterns, blood pressure, and type 2 diabetes mellitus (T2DM) risk in elderly Chinese males, using unsupervised machine learning techniques and SHAP-based model interpretation. We identified four distinct dietary patterns—“high-fiber nutrient-dense,” “staple–protein,” “seafood-eggs,” and “sugary and processed foods”—and examined their impact, alongside obesity and blood pressure, on the prevalence and risk of T2DM. Furthermore, the results remained similar even when sensitivity analyses were performed. The findings provide important insights into modifiable risk factors and their role in predicting diabetes outcomes.

### 4.1 Type 2 diabetes mellitus prevalence and clinical characteristics

Among elderly males in our study, the prevalence of type 2 diabetes mellitus was substantial, affecting 52% of participants, substantially exceeding the reported national prevalence among Chinese adults aged ≥60 years, which ranges from ~20 to 25% (38). Interestingly, despite the high prevalence of type 2 diabetes, no significant differences were observed in BMI, waist circumference, blood pressure, lipid profile, or other metabolic markers between participants with and without diabetes. This finding may reflect the relatively homogeneous characteristics of this elderly population, many of whom may have adopted long-term lifestyle modifications or health management strategies that attenuate traditional risk factors (39, 40). Furthermore, unmeasured factors—such as habitual dietary patterns, genetic predisposition, or ongoing medication use—could contribute to diabetes risk and partially explain the absence of significant differences (41, 42). Comparable results have been reported in other older populations, indicating that standard anthropometric and metabolic markers may have limited utility to discriminate diabetes risk among elderly individuals (30, 43).

### 4.2 Machine learning-based dietary pattern classification

To overcome the limitations of conventional dietary pattern analyses, we employed UMAP (Uniform Manifold Approximation and Projection) to reduce dimensionality and cluster high-dimensional dietary intake data (19). This unsupervised approach effectively identified four distinct dietary patterns: “high-fiber nutrient-dense,” “staple–protein,” “seafood–eggs,” and “sugary and processed foods,” demonstrating the capacity of machine learning methods to uncover latent dietary structures without predefined assumptions. The high-fiber nutrient-dense pattern was characterized by high intake of plant-based and nutrient-rich foods, whereas the sugary and processed foods pattern was characterized by high consumption of processed and high-sugar items, reflecting contrasting dietary behaviors. The “staple–protein” and “seafood–eggs” patterns occupied intermediate positions and reflected regionally or culturally typical dietary habits. These four dietary patterns accounted for 6.07%−12.33% of the variance in dietary intake individually, and cumulatively explained up to 39.10% of the total variance. Although the percentage of variance explained by individual clusters may appear modest, this is consistent with previous studies on dietary patterns, where typical variance explained ranges from 5 to 30% due to the inherent complexity and heterogeneity of dietary behaviors (44, 45). Therefore, the identified patterns capture meaningful distinctions in dietary habits among elderly Chinese men and provide a robust basis for examining associations with obesity and type 2 diabetes risk.

### 4.3 Associations between dietary patterns, obesity, and T2DM

Our results demonstrate that elderly males adhering to dietary patterns with lower nutritional quality are at higher risk of obesity (46). The sugary and processed foods pattern had the strongest association with obesity, suggesting that diets high in refined sugars and processed items may drive adiposity more than traditional staple-based or seafood-rich patterns (47, 48). These findings align with prior studies indicating that nutrient-dense, high-fiber diets are protective against excessive weight gain, whereas energy-dense, nutrient-poor diets promote metabolic dysregulation (49). The robustness of these associations after adjusting for sociodemographic, lifestyle, biochemical, and clinical factors underscores the independent role of dietary quality in obesity risk (50).

All non-reference dietary patterns were significantly associated with higher T2DM prevalence compared with the high-fiber nutrient-dense pattern. The staple–protein, seafood–eggs, and sugary and processed foods patterns showed consistent positive associations across fully adjusted models. Unhealthy dietary patterns were associated with impaired glycemic regulation in this cross-sectional analysis, potentially through mechanisms such as increased insulin resistance, chronic inflammation, or unfavorable lipid profiles (50, 51). Sensitivity analyses further indicated that these associations were largely independent of total energy intake, emphasizing that the type and combination of foods consumed may exert a stronger influence on T2DM risk than overall caloric load (52, 53).

Stratified analyses revealed that T2DM prevalence varied across dietary clusters and adiposity levels, indicating potential interactions between diet and body fat in influencing diabetes risk (54). Although overall blood pressure did not differ significantly between diabetic and non-diabetic participants, it may still serve as a modifier in diabetes pathophysiology through sex-specific hormonal or vascular mechanisms (55, 56). The inverse association observed between obesity and T2DM risk may reflect survivor bias, age-related changes in fat distribution, or metabolic heterogeneity among older adults, highlighting the complexity of risk factor interactions in this demographic (57, 58). Our sensitivity analyses demonstrated that the associations between dietary patterns, obesity, and T2DM risk persisted after accounting for BMI stratification and caloric intake, indicating the internal stability and robustness of the findings.

### 4.4 Feature importance and interpretability using SHAP

To enhance model transparency and understand the predictive contributions of individual features, we employed SHAP values (59). SHAP analysis indicated that dietary patterns, total energy intake, and physical activity were key predictors in the model, with dietary patterns exhibiting the greatest overall contribution. Their effects were mainly negative, implying that optimizing these factors may offer protective benefits against diabetes mellitus. This result is consistent with previous studies, indicating that adherence to certain dietary patterns may play a beneficial role in the prevention and management of T2DM (60–63). The use of the shapviz package allowed for a more nuanced understanding of how each variable contributed to individual model predictions (64). Variables displayed in red were considered indicative of protective effects, while cumulative plots provided a multi-layered view of predicted risk. Such interpretability is essential for translating complex outputs from machine learning models into practical insights for clinical decision-making and public health planning.

### 4.5 Public health implications and recommendations

These findings have several important implications for dietary guidance, health education, and strategies for the prevention of type 2 diabetes mellitus (T2DM). First, the high prevalence of diabetes, despite the absence of significant differences in conventional risk markers, suggests that dietary patterns provide additional predictive value that could complement existing clinical screening tools. Second, the application of interpretable machine learning techniques, such as UMAP and SHAP, represents a promising approach for advancing precision nutrition and personalized prevention strategies.

Public health interventions should focus on promoting nutrient-rich dietary habits and culturally appropriate food choices, emphasizing increased consumption of vegetables, whole grains, and seafood, while limiting processed and sugary foods.

### 4.6 Strengths and limitations

This study has several key strengths. First, it draws on a large, well-characterized cohort of elderly Chinese males, enabling robust analyses of dietary patterns, obesity, blood pressure, and T2DM risk. Second, the application of advanced analytical approaches, including unsupervised machine learning (UMAP) combined with factor analysis, allowed for the identification of distinct dietary patterns and the effective visualization of complex multidimensional dietary behaviors. Third, the use of SHAP values within an XGBoost modeling framework provided interpretable insights into the relative contributions of demographic, lifestyle, and dietary factors to T2DM risk, enhancing the translational relevance of our findings for public health interventions. Finally, comprehensive multivariable logistic regression models with extensive covariate adjustment and sensitivity analyses support the validity and reliability of the observed associations between dietary patterns, obesity, and T2DM risk.

Nonetheless, several limitations should be acknowledged. First, the cross-sectional design prevents causal inferences regarding the relationships between dietary patterns, obesity, and T2DM, limiting conclusions about temporal or mechanistic pathways. Second, dietary intake was assessed through self-reported measures, which may be prone to recall bias or misreporting, particularly in an older population. Third, since the study focused exclusively on elderly Chinese males, the generalizability of these findings to women or to other ethnic and age groups may be limited. Dietary behaviors, metabolic responses, and T2DM risk profiles can differ by sex due to hormonal, physiological, and sociocultural factors. Similarly, other ethnic groups may exhibit distinct dietary patterns, obesity prevalence, or genetic predispositions affecting T2DM risk. Future studies including women and diverse populations are needed to assess whether the identified dietary patterns and their associations with obesity and T2DM risk are consistent across broader demographic groups. Fourth, residual confounding from unmeasured variables, such as genetic predispositions or detailed nutrient metabolism data, cannot be entirely excluded. Fifth, this study did not include an independent external validation dataset due to cohort constraints. While sensitivity analyses served as an internal robustness assessment, future research should validate these findings in independent prospective cohorts and other populations to enhance generalizability and clinical applicability. Finally, although advanced machine learning techniques were employed, the complexity of these models may restrict their immediate application in routine clinical practice without further validation.

Overall, the combination of rigorous statistical modeling, interpretable machine learning analyses, and detailed dietary pattern characterization constitutes a major methodological strength, whereas the observational design and reliance on self-reported dietary data remain important limitations to be addressed in future longitudinal studies.

## 5 Conclusions

In this study of elderly Chinese males, we observed a high prevalence of newly diagnosed Type 2 diabetes mellitus (T2DM), with nearly half of participants affected. Contrary to traditional assumptions, obesity was inversely associated with T2DM risk, even after adjustment for a wide range of demographic, lifestyle, and biochemical covariates. Dietary patterns emerged as key determinants of both obesity and T2DM risk. Specifically, adherence to unhealthy dietary patterns—namely staple–protein, seafood–eggs, and sugary and processed foods—was consistently associated with an increased risk of obesity and T2DM, while a high-fiber nutrient-dense diet was protective. Machine learning analyses further highlighted the prominent role of dietary behaviors, total energy intake, and physical activity as influential predictors of T2DM risk. These findings suggest that, beyond traditional anthropometric measures, diet quality and lifestyle factors play a critical role in shaping metabolic health in this population. Intervention strategies focusing on promoting nutrient-dense diets and modifiable lifestyle behaviors may be effective in reducing the burden of obesity and T2DM among elderly Chinese males. Importantly, this study is among the first to leverage unsupervised machine learning with SHAP analysis to uncover dietary and metabolic determinants of T2DM in elderly Chinese men, offering novel insights for precision prevention strategies.

## Data Availability

The raw data supporting the conclusions of this article will be made available by the authors, without undue reservation.
