# Correction: Associations of dietary patterns and obesity with type 2 diabetes in elderly Chinese men: a machine learning approach

**DOI:** 10.3389/fnut.2026.1882361

**Published:** 2026-08-07

**Authors:** Haowei Sun, Lijin Zhu, Peng Wang, Keqing Yuan, Saida Salima Nawrin, Yufei Cui, Longfei Li

**Affiliations:** 1 College of Physical Education and Health, Heze University, Heze, Shandong, China; 2 Graduate School of Medicine, Tohoku University, Sendai, Japan

**Keywords:** type 2 diabetes mellitus, dietary patterns, obesity, unsupervised machine learning, SHAP analysis

The title of this article was erroneously given as: Dietary patterns and obesity are associated with type 2 diabetes risk in elderly Chinese men: a machine learning approach]. The correct title of the article is “Associations of dietary patterns and obesity with type 2 diabetes in elderly Chinese men: a machine learning approach”.

There was a mistake in Figures 1, 2, Tables 1, 3–5, **Supplementary Tables S1–S6**, and **Supplementary Figures S1, S2** as published. There was an error in the preprocessing of variables used in the supervised machine learning model. The corrected Figures 1, 2 and Tables 1, 3–5 and their captions appear below. The corrected **Supplementary Tables S1–S6, and Supplementary Figures S1, S2** have been published.

**Figure 1:**
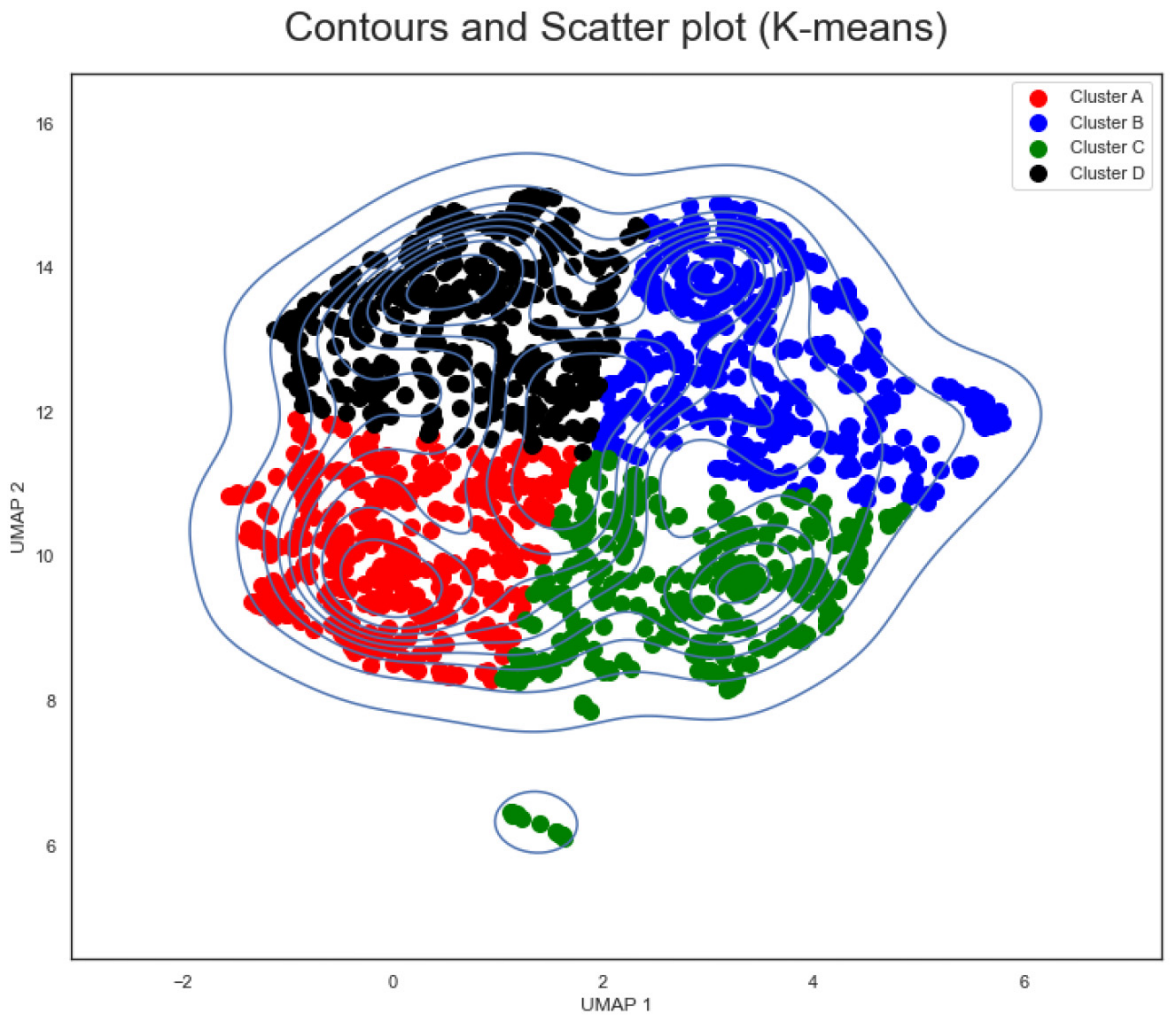
Scatter plot and contour map. The data has been reduced to two dimensions [Dimension 1 (D1) and Dimension 2 (D2)] using UMAP. Each point on the graph represents an individual participant in the study. The contour lines are curves that show areas of constant Gaussian values in the 2D space, connecting points of similar 2D values. Number clusters (*n* = 4) were chosen optimally by contour map analysis. Colors indicate cluster assignment using *K*-means clustering (*K* = 4; see Section 2 for details).

**Figure 2:**
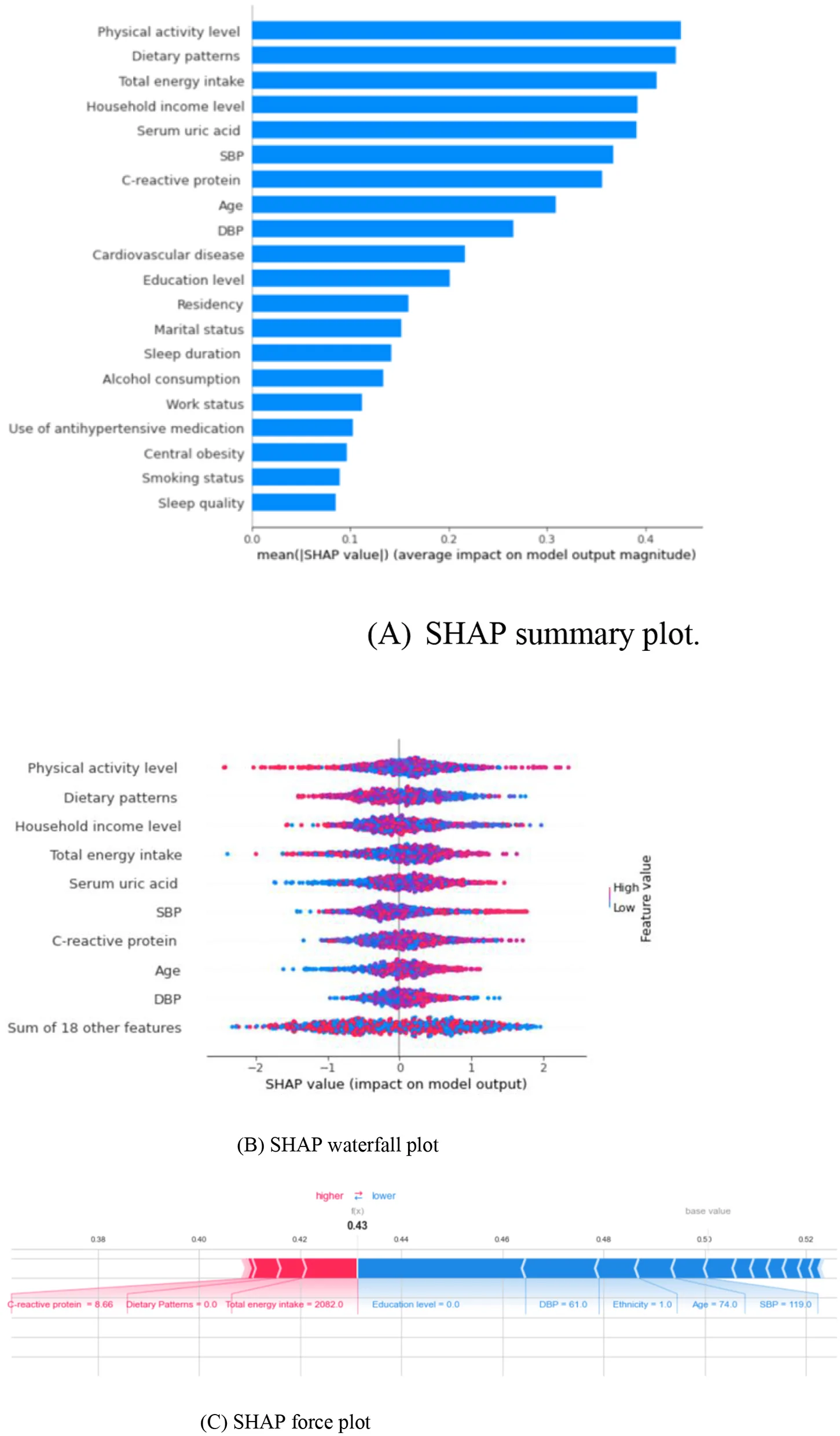
SHAP values of dietary patterns for T2DM model. **(A)** SHAP summary plot. Absolute feature contribution of the 20 features with the highest mean absolute SHAP value. **(B)** SHAP waterfall plot. Complete distribution of the SHAP values for the top 20 features based on the highest mean absolute SHAP value. Each sample of the test set is represented as a data point per feature, and the *x* axis shows the positive or negative effect on the model's prediction of the feature. The color coding depicts the value of the feature and is scaled independently based on the range observed in the data. **(C)** SHAP force plot. Representative force plots, depicting how the features contributed to the prediction for specific data points.

**Table 1:** Demographic characteristics of the diabetes.

Characteristics	Male		
	T2DM status	*P*-value	
	**No (471, 48%)**	**Yes (511, 52%)**	
Age	74.21 ± 8.65	74.48 ± 8.78	0.624
BMI	26.85 ± 4.75	26.67 ± 4.89	0.562
Hip circumference	104.86 ± 14.52	105.32 ± 14.51	0.625
Waist circumference	94.40 ± 14.89	95.19 ± 14.85	0.722
Physical activity	1556.02 ± 846.16	1498.43 ± 865.29	0.293
SBP	136.63 ± 26.12	133.97 ± 26.21	0.112
DBP	84.91 ± 14.71	85.29 ± 14.00	0.684
Income	5416.94 ± 2560.97	5428.83 ± 2637.67	0.594
Serum insulin	13.69 ± 6.60	13.76 ± 6.80	0.874
Homa-IR	3.27 ± 1.63	3.20 ± 1.59	0.508
HDL	60.27 ± 16.79	60.15 ± 17.11	0.908
LDL	126.00 ± 42.62	124.06 ± 43.21	0.479
Triglycerides	167.38 ± 71.75	177.67 ± 72.53	0.126
CRP	5.09 ± 2.84	5.11 ± 2.87	0.963
Uric acid	5.49 ± 1.45	5.57 ± 1.45	0.439
Total energy intake	2467.25 ± 573.65	2532.60 ± 562.92	0.072
hbA1c	5.32 ± 1.68	8.46 ± 1.45	0.001
Fasting blood glucose	136.48 ± 37.42	133.97 ± 36.70	0.039
Educational level (*N*, %)			
Illiteracy	114 (24.2%)	138 (27.0%)	0.106
Primary school	106 (22.5%)	138 (27.0%)	
Middle school	127 (27.0%)	128 (25.0%)	
College or above	124 (26.3%)	107 (20.9%)	
Smoking status (*N*, %)			
Never	172 (36.5%)	167 (32.7%)	0.248
Former	165 (35.0%)	175 (34.2%)	
Current	134 (28.5%)	169 (33.1%)	
Alcohol consumption (*N*, %)			
No	66 (14.0%)	85 (16.6%)	0.564
Drinker	405 (86.0%)	426 (83.4%)	
Use of antidiabetic medication (*N*, %)			
No	235 (49.9%)	249 (48.7%)	0.034
Yes	236 (50.1%)	262 (51.3%)	
Family history of diabetes (*N*, %)			
No	231 (49.0%)	256 (50.1%)	0.742
Yes	240 (51.0%)	255 (49.9%)	
Family history of hypertension (*N*, %)			
No	266 (56.5%)	250 (49.1%)	0.018
Yes	205 (43.5%)	261 (51.1%)	
Sleep duration (*N*, %)			
< 7 h	234 (49.7%)	261 (51.1%)	0.866
7–9 h	169 (35.7%)	174 (34.1%)	
>9 h	69 (14.6%)	76 (14.9%)	
Sleep quality (*N*, %)			
Poor	164 (34.8%)	157 (30.7%)	0.171
Good	307 (65.2%)	354 (69.3%)	
Marital status (*N*, %)			
Other	239 (50.7%)	272 (53.2%)	0.720
Married	232 (49.3%)	239 (46.8%)	
Work status (*N*, %)			
Unemployed	136 (28.9%)	163 (31.9%)	0.566
Employed	335 (71.1%)	348 (68.1%)	
Residency (*N*, %)			
Rural area	205 (51.4%)	248 (50.9%)	0.876
Urban area	229 (48.6%)	251 (49.1%)	
General obesity (*N*, %)			
No	229 (48.6%)	240 (47.0%)	0.604
Yes	242 (51.4%)	271 (53.0%)	
Central obesity (*N*, %)			
No	256 (54.4%)	253 (49.5%)	0.129
Yes	215 (45.6%)	258 (50.5%)	
Ethnicity (*N*, %)			
Minority	239 (50.7%)	252 (49.3%)	0.655
Han Chinese	232 (49.3%)	259 (50.7%)	
Cardiovascular disease (*N*, %)			
No	237 (50.3%)	255 (49.9%)	0.8963
Yes	234 (49.7%)	256 (50.1%)	

This table presents the demographic data, blood test results, and clinical characteristics related to diabetes symptoms. Group comparisons were performed using Chi-square tests or analysis of variance (ANOVA), with means and standard deviations (mean ± SD) shown in parentheses. *P* values are derived from one-way ANOVA or Chi-square tests, as appropriate. BMI, body mass index; SBP, systolic blood pressure; DBP, diastolic blood pressure; FBG, fasting blood glucose; RBC, red blood cells; HB, hemoglobin; WBC, white blood cells; PA, physical activity; LDL-C, low-density lipoprotein cholesterol; HDL-C, high-density lipoprotein cholesterol.

**Table 3:** Multivariable logistic regression analysis of the association between obesity and type 2 diabetes mellitus.

Model	Obeslty	T2DM	*P*-value
Model 1	1 (ref)	0.268 (0.081, 0.458)	0.012
Model 2	1 (ref)	0.271 (0.084, 0.469)	0.014
Model 3	1 (ref)	0.275 (0.083, 0.477)	0.015
Model 4	1 (ref)	0.278 (0.084, 0.474)	0.015
Model 5	1 (ref)	0.278 (0.083, 0.475)	0.015

Model 1: crude model; Model 2: adjust for age, smoking status, marital status, work status, residency, ethnicity, household income level, alcohol consumption, physical activity, sleep duration; Model 3: adjust for blood pressure, HDL, LDL, triglycerides, CRP, serum uric acid; Model 4: adjust for cluster; Model 5: adjust for family history of hypertension, family history of diabetes, use of antihypertensive medication.

**Table 4:** Multivariable logistic regression analysis of the association between dietary patterns and obesity.

Model	Cluster A (high-fiber nutrient-dense)	Cluster B (staple–protein)	Cluster C (seafood–eggs)	Cluster D (sugary and processed foods)	*P*-value
Model 1	1 (ref)	0.351 (0.119, 0.585)	0.414 (0.181, 0.648)	0.610 (0.368, 0.831)	0.003
Model 2	1 (ref)	0.368 (0.148, 0.589)	0.433 (0.190, 0.654)	0.616 (0.381, 0.848)	0.001
Model 3	1 (ref)	0.390 (0.166, 0.618)	0.438 (0.207, 0.669)	0.630 (0.410, 0.856)	0.001
Model 4	1 (ref)	0.362 (0.146, 0.592)	0.431 (0.169, 0.648)	0.590 (0.370, 0.824)	0.002
Model 5	1 (ref)	0.360 (0.118, 0.569)	0.386 (0.109, 0.613)	0.583 (0.358, 0.864)	0.002

Model 1: crude model; Model 2: adjust for age, smoking status, marital status, work status, residency, ethnicity, household income level, alcohol consumption, physical activity, sleep duration; Model 3: adjust for fasting blood glucose, HbA1c, serum insulin, HDL, LDL, triglycerides, CRP, serum uric acid; Model 4: adjust for family history of hypertension, family history of diabetes, use of antihypertensive medication.

**Table 5:** Multivariable logistic regression analysis of the association between dietary patterns and type 2 diabetes mellitus.

Model	Cluster A (high-fiber nutrient-dense)	Cluster B (staple–protein)	Cluster C (seafood–eggs)	Cluster D (sugary and processed foods)	*P*-value
Model 1	1 (ref)	1.321 (1.041, 1.644)	1.268 (1.013, 1.621)	1.546 (1.213, 1.977)	0.012
Model 2	1 (ref)	1.341 (1.072, 1.651)	1.256 (1.018, 1.559)	1.482 (1.164, 1.925)	0.006
Model 3	1 (ref)	1.376 (1.091, 1.712)	1.276 (1.046, 1.603)	1.460 (1.123, 1.862)	0.008
Model 4	1 (ref)	1.388 (1.122, 1.768)	1.317 (1.066, 1.664)	1.455 (1.118, 1.854)	0.006
Model 5	1 (ref)	1.412 (1.112, 1.765)	1.336 (1.084, 1.757)	1.494 (1.106, 1.884)	0.005

Model 1: crude model; Model 2: adjust for age, BMI, smoking status, marital status, work status, residency, ethnicity, household income level, alcohol consumption, physical activity, sleep duration; Model 3: adjust for HDL, LDL, triglycerides, CRP, serum uric acid; Model 4: adjust for blood pressure; Model 5: adjust for family history of hypertension, family history of diabetes, use of antihypertensive medication.

The funder, Shandong Provincial Natural Science Foundation, ZF2024QF221 to Longfei Li, was erroneously omitted. The correct **Funding** statement appears below.

**Funding**

The author(s) declared that financial support was received for this work and/or its publication. This work was supported by Shandong Provincial Natural Science Foundation under the grant number, ZF2024QF22, to Longfei Li.

A correction has been made to the section **Abstract**, *Results*, the incorrect sentence was written as, “The prevalence of newly diagnosed T2DM in males was 48.37%. Obesity was inversely associated with T2DM risk across all models (odds ratios: 0.272–0.278, all *P* < 0.05).” The correct sentence should read as, “The prevalence of T2DM in males was 52%. Obesity was inversely associated with T2DM odds across all models (odds ratios: 0.268–0.278, all *P* < 0.05).”

A correction has been made to the section **Abstract**, *Results*, the incorrect sentence was written as, “Shapley Additive Explanations (SHAP) analysis highlighted dietary behaviors, total energy intake, and physical activity as major contributors to T2DM prediction.” The correct sentence should read as, “Shapley additive explanations (SHAP) analysis highlighted, physical activity level and dietary patterns as major contributors to T2DM prediction.”

A correction has been made to the **Section 2**, *Section 2.2*. The incorrect sentence was written as, “General obesity was further defined as BMI ≥28 kg/m^2^, based on the criteria recommended by the Working Group on Obesity in China (WGOC) (28).” The correct sentence should read as, “Obesity was further defined as BMI ≥28 kg/m^2^, based on the criteria recommended by the Working Group on Obesity in China (WGOC) (28).”

A correction has been made to the **Section 2**, *Section 2.6*, paragraph 1. The incorrect paragraph was written as, “To identify dietary patterns, Uniform Manifold Approximation and Projection (UMAP) was applied in conjunction with varimax rotation (37). Dimensionality reduction was first conducted using UMAP, followed by the extraction of latent features through factor analysis based on the raw dataset. The UMAP parameters were set as follows: umap_min_dist = 0.1, max_components = 2, n_neighbors = 10, and Chebyshev distance as the metric. These values were optimized through systematic parameter tuning to ensure the generation of distinct and well-separated clusters.” The correct paragraph should read as, “To identify dietary patterns, an unsupervised machine-learning framework combining nonlinear dimensionality reduction, clustering, and factor analysis was applied. Dietary intake data derived from the validated FFQ were first aggregated into 21 predefined food groups and standardized prior to analysis. UMAP was not used for variance decomposition or factor extraction. Uniform manifold approximation and projection (UMAP) was employed solely as a nonlinear dimensionality-reduction and visualization tool to project the high-dimensional dietary intake data into a low-dimensional space while preserving local neighborhood structure. UMAP was not used for variance decomposition or factor extraction. The UMAP parameters were specified a priori as follows: n_neighbors = 10, min_dist = 0.1, n_components = 2, and Chebyshev distance as the similarity metric (37). These values were optimized through systematic parameter tuning to ensure the generation of distinct and well-separated clusters.”

A correction has been made to the **Section 2**, *Section 2.6*, paragraph 2. The incorrect paragraph read as, “To determine the optimal number of clusters, 2D data were applied to *K*-means clustering; the default settings were used for *K*-means clustering. The number of clusters was determined using the elbow method. The number of clusters was determined using the elbow method and the final cluster number was also confirmed as peak numbers in the contour map. We applied the elbow method on the UMAP-reduced features.” The correct paragraph should read as, “Clustering was subsequently performed using the *K*-means algorithm applied to the two-dimensional UMAP embedding. The optimal number of clusters (*K*) was initially explored using the elbow method based on within-cluster sum of squares calculated in the UMAP-reduced space. As a complementary, descriptive aid, kernel density contour maps were visually inspected to assess the distribution of observations in the two-dimensional space; however, final cluster selection was primarily guided by the elbow criterion and interpretability. *K*-means clustering was implemented using default initialization and convergence settings. To assess the robustness of dietary pattern clusters derived from *K*-means on the UMAP embedding, we generated 500 bootstrap samples of the original dataset, applied *K*-means clustering with the same number of clusters and UMAP parameters to each sample, and quantified cluster stability as the average proportion of times participants were assigned to the same cluster across all iterations. Silhouette scores were computed in the original feature space, indicating moderate cluster cohesion and separation across the derived dietary patterns.”

A correction has been made to the **Section 2**, *Section 2.6*, paragraph 3. The incorrect paragraph read as, “The two-dimensional representations produced by UMAP were then subjected to factor analysis with varimax rotation, and the resulting factors were interpreted based on their loadings on the 21 predefined food groups. Cluster labeling was performed according to the relative contributions of each food group to the factors, with “trend values” reflecting the standardized factor scores across clusters.” The correct paragraph should read as, “To characterize the dietary composition underlying each cluster, exploratory factor analysis with varimax rotation was conducted on the original 21 food-group variables, rather than on the UMAP-reduced dimensions. Factor analysis was used exclusively for *post hoc* interpretation of dietary patterns and not for cluster construction. Factor loadings with absolute values ≥0.25 were considered meaningful contributors to each pattern. Dietary clusters were subsequently labeled based on the dominant food groups reflected in the factor-loading structure. Standardized factor scores were summarized descriptively across clusters to illustrate relative dietary tendencies.”

A correction has been made to the **Section 2**, *Section 2.6*, paragraph 4. The incorrect paragraph read as, “To complement the unsupervised identification of dietary patterns, a supervised classification model was subsequently applied to evaluate disease risk. Specifically, the dietary clusters derived from the UMAP–*K*-means procedure were incorporated as input features—together with metabolic and lifestyle variables—into an XGBoost classifier trained to predict Type 2 diabetes mellitus (T2DM) status. This hybrid analytical framework enabled both data-driven discovery of dietary structures and quantitative assessment of their predictive relevance for T2DM. The interpretability of the model was further enhanced by applying SHAP, which quantified the relative contribution of each feature to predicted T2DM risk.” The correct paragraph should read as, “To complement the unsupervised identification of dietary patterns, a supervised machine-learning approach was applied to evaluate their association with Type 2 diabetes mellitus (T2DM). Specifically, dietary cluster membership derived from the UMAP–*K*-means procedure was included as an exposure variable, together with selected demographic, anthropometric, and lifestyle covariates, in an extreme gradient boosting (XGBoost) classifier. This combined analytical strategy enabled data-driven identification of dietary profiles and exploratory assessment of their predictive relevance for T2DM.”

A correction has been made to the **Section 2**, *Section 2.6*, paragraph 5. The incorrect paragraph reads as, “To evaluate the relative contribution of various features to Type 2 diabetes mellitus risk, we trained an XGBoost classifier using the study dataset. Model performance was assessed using 5-fold cross-validation. Hyperparameters were optimized via grid search to maximize predictive performance. To interpret the contribution of individual features to T2DM risk, we applied SHAP (SHapley Additive exPlanations) analysis using the shap-viz package. SHAP values quantify the impact of each feature on the model's predictions, allowing for ranking of the most influential variables. Force plots and summary plots were generated to visualize how dietary patterns, energy intake, and lifestyle factors influence T2DM risk, thereby enhancing interpretability and providing insight into potential mechanisms linking these factors to disease onset.” The correct paragraph should read as, “Model performance was evaluated using five-fold cross-validation, and hyperparameters were optimized through grid search. To enhance interpretability, SHapley Additive exPlanations (SHAP) were computed to quantify the relative contribution of each predictor to the model output. SHAP summary plots and force plots were generated to provide local, individual-level explanations of model predictions, illustrating how each feature contributed to deviations from the baseline risk. Categorical variables were transformed into binary dummy variables using one-hot encoding prior to model training. Consequently, SHAP force plots reflect the contribution of category membership rather than any ordinal numerical effect.”

A correction has been made to the **Section 3**, *Section 3.2*, paragraph 2. The incorrect paragraph reads as, “The data has been reduced to two dimensions [Dimension 1 (D1) and Dimension 2 (D2)] using UMAP. Each point on the graph represents an individual participant in the study. The contour lines are curves that show areas of constant Gaussian values in the 2D space, connecting points of similar 2D values. Number clusters (*n* = 4) were chosen optimally by contour map analysis. Colors indicate cluster assignment using K-means clustering (*K* = 4; see “Section 2” for details).” The correct paragraph should read as, “Cluster robustness was assessed using silhouette scores in the standardized original feature space, with 500 bootstrap resamples used to compute 95% confidence intervals. As shown in **Supplementary Table S7**, the highest mean score was observed for *K* = 4 (0.46, 95% CI: 0.43–0.49). Scores for *K* = 3 and *K* = 5 were lower (0.36 and 0.39), indicating that *K* = 4 is the optimal cluster number. Analyses across different UMAP parameter settings (n_neighbors = 10–50, min_dist = 0.1–0.5) and alternative *K* values confirmed that the clustering structure remained stable.”

A correction has been made to the **Section 3**, *Section 3.4*, paragraph 1. The incorrect sentence was written as, “Notably, factors such as dietary behaviors, total energy consumption, and physical activity levels were identified as the most prominent negative predictors.” The correct sentence should read as, “Notably, physical activity level and dietary patterns were identified as the primary influential factors in the model predictions, showing the most substantial contributions among the examined features.”

A correction has been made to the **Section 3**, *Section 3.1*, paragraph 1. The incorrect sentence was written as, “As shown in Table 1, 48.37% of the male participants were newly diagnosed with Type 2 diabetes mellitus.” The correct sentence should read as, “As shown in Table 1, 52% of the male participants had Type 2 diabetes mellitus.”

A correction has been made to the **Section 3**, *Section 3.5*, paragraph 1. The incorrect paragraph was written as, “Table 3 displays the outcomes of five sequential logistic regression models assessing the link between obesity and the risk of Type 2 diabetes mellitus (DM) based on a cross-sectional analysis. Throughout all models, obesity was consistently found to be inversely related to the risk of T2DM, as evidenced by odds ratios between 0.272 and 0.278, all with *P*-values under 0.05. This negative association remained robust even with the addition of covariates in each subsequent model.” The correct paragraph should read as, “Table 3 displays the outcomes of five sequential logistic regression models assessing the link between obesity and the risk of Type 2 diabetes mellitus (DM) based on a cross-sectional analysis. Throughout all models, obesity was consistently found to be inversely related to the risk of T2DM, as evidenced by odds ratios between 0.268 and 0.278, all with *P*-values under 0.05. This negative association remained robust even with the addition of covariates in each subsequent model.”

A correction has been made to the **Section 3**, *Section 3.5*, paragraph 2. The incorrect paragraph was written as, “In the multivariable logistic regression analysis, dietary patterns were significantly associated with obesity risk among elderly Chinese males (Table 4). Using the high-fiber nutrient-dense dietary pattern as the reference, participants adhering to the staple–protein pattern exhibited a higher risk of obesity across all models (Model 1: β = 0.352, 95% CI: 0.120–0.584, *P* = 0.002; Model 5: β = 0.348, 95% CI: 0.128–0.568, *P* = 0.003). Similarly, the seafood–eggs pattern was positively associated with obesity (Model 1: β = 0.415, 95% CI: 0.182–0.648, *P* = 0.002; Model 5: β = 0.386, 95% CI: 0.159–0.613, *P* = 0.003). Notably, the sugary and processed foods pattern demonstrated the strongest association with obesity risk, with consistently significant effects after full adjustment (Model 1: β = 0.601, 95% CI: 0.372–0.830, *P* = 0.002; Model 5: β = 0.581, 95% CI: 0.358–0.804, *P* = 0.003). These associations remained robust after adjusting for a wide range of sociodemographic, lifestyle, biochemical, and clinical covariates, suggesting that dietary patterns characterized by lower nutritional quality are independently linked to increased obesity risk in this population.” The correct paragraph should read as, “In the multivariable logistic regression analysis, dietary patterns were significantly associated with obesity odds among elderly Chinese males (Table 4). Using the high-fiber nutrient-dense dietary pattern as the reference, participants adhering to the staple–protein pattern exhibited a higher odds of obesity across all models (Model 1: β = 0.351, 95% CI: 0.119–0.585, *P* = 0.003; Model 5: β = 0.360, 95% CI: 0.118–0.569, *P* = 0.002). Similarly, the seafood–eggs pattern was positively associated with obesity (Model 1: β = 0.414, 95% CI: 0.181–0.648, *P* = 0.003; Model 5: β = 0.386, 95% CI: 0.109–0.613, *P* = 0.002). Notably, the sugary and processed foods pattern demonstrated the strongest association with obesity odds, with consistently significant effects after full adjustment (Model 1: β = 0.610, 95% CI: 0.368–0.831, *P* = 0.003; Model 5: β = 0.583, 95% CI: 0.358–0.864, *P* = 0.002). These associations remained robust after adjusting for a wide range of sociodemographic, lifestyle, biochemical, and clinical covariates, suggesting that dietary patterns characterized by lower nutritional quality are independently linked to increased obesity odds in this population.”

A correction has been made to the **Section 3**, *Section 3.5*, paragraph 3. The incorrect paragraph was written as, “As shown in Table 5, all three non-reference dietary patterns were significantly associated with increased odds of type 2 diabetes mellitus (T2DM) compared with the high-fiber nutrient-dense pattern. In Model 1, ORs were 1.32 (95% CI: 1.04–1.64) for staple–protein, 1.26 (95% CI: 1.01–1.62) for seafood–eggs, and 1.54 (95% CI: 1.21–1.97) for sugary and processed foods, with *P* = 0.012. These associations remained significant across Models 2–5 after progressively adjusting for demographic factors, lifestyle behaviors, metabolic biomarkers, blood pressure, T2DM status, and family history or medication use (all *P* < 0.01), indicating a consistent positive relationship between unhealthy dietary patterns and T2DM odds in this population.” The correct paragraph should read as, “As shown in Table 5, all three non-reference dietary patterns were significantly associated with increased odds of type 2 diabetes mellitus (T2DM) compared with the high-fiber nutrient-dense pattern. In Model 1, ORs were 1.32 (95% CI: 1.04–1.64) for staple–protein, 1.26 (95% CI: 1.01–1.62) for seafood–eggs, and 1.54 (95% CI: 1.21–1.97) for sugary and processed foods, with *P* = 0.012. These associations remained significant across Models 2–5 after progressively adjusting for demographic factors, lifestyle behaviors, metabolic biomarkers, blood pressure, T2DM status, and family history or medication use (all *P* < 0.01), indicating a consistent positive relationship between unhealthy dietary patterns and T2DM odds in this population.”

A correction has been made to the **Section 4**, *Section 4.1*, paragraph 1. The incorrect sentence was written as, “The overall prevalence of newly diagnosed type 2 diabetes mellitus among elderly males was notably high (48.37%), substantially exceeding the reported national prevalence among Chinese adults aged ≥60 years, which ranges from ~20 to 25% (38).” The correct sentence reads as, “Among elderly males in our study, the prevalence of type 2 diabetes mellitus was substantial, affecting 52% of participants, substantially exceeding the reported national prevalence among Chinese adults aged ≥60 years, which ranges from ~20 to 25% (38).”

The original version of this article has been updated.

