# Supplementary material for: Dietary patterns and obesity are associated with type 2 diabetes risk in elderly Chinese men: a machine learning approach
Source: Front Nutr. 2025 Nov 26;12:1705683. doi: 10.3389/fnut.2025.1705683 (PMC12689295; doi:10.3389/fnut.2025.1705683)
Supplement: Supplementary file 1 [file Table_1.docx]

**Supplementary Materials**

**Table 1.** Multivariable Logistic Regression Analysis of the Association Between Obesity and Type 2 diabetes mellitus

|  | | **DM** | | | **P** |
| --- | --- | --- | --- | --- | --- |
| Model 1 | 1 (Ref) | | 0.272(0.086,0.460) | 0.013 | |
| Model 2 | 1 (Ref) | | 0.275(0.084,0.467) | 0.014 | |
| Model 3 | 1 (Ref) | | 0.277(0.083,0.473) | 0.015 | |
| Model 4 | 1 (Ref) | | 0.278(0.084,0.474) | 0.015 | |
| Model 5 | 1 (Ref) | | 0.278(0.083,0.475) | 0.014 | |
| Model 6 | 1 (Ref) | | -0.125(-0.005,0.255) | 0.138 | |

Model 1: adjust obesity; Model 2:adjust age, BMI, smoking status, marital status, work status, residency, ethnicity, household income level, Alcohol consumption, physical activity, sleep duration; Model 3: adjust use of fasting blood glucose, HbA1, serum insulin, HDL, LDL, triglycerides, CRP, serum uric acid; Model 4: adjust cluster, general obesity; Model 5: adjust, family history of hypertension, family history of diabetes, use of antihypertensive medication, use of antidiabetic medication; Model 6: adjust total caloric intake.

**Table 2.** Multivariable Logistic Regression Analysis of the Association Between Dietary Patterns and Obesity

|  | Cluster A  (High-fiber nutrient-dense) | Cluster B  (Staple–protein) | Cluster C  (Seafood–eggs) | Cluster D  (Sugary and processed foods) | P-value |
| --- | --- | --- | --- | --- | --- |
| Model 1 | 1 (Ref) | 0.352(0.120,0.584) | 0.415(0.182,0.648) | 0.601(0.372,0.830) | 0.002 |
| Model 2 | 1 (Ref) | 0.371(0.145,0.597) | 0.422(0.190,0.654) | 0.618(0.390,0.846) | 0.001 |
| Model 3 | 1 (Ref) | 0.389(0.165,0.613) | 0.438(0.207,0.669) | 0.629(0.402,0.858) | 0.001 |
| Model 4 | 1 (Ref) | 0.362(0.140,0.584) | 0.401(0.170,0.632) | 0.597(0.372,0.822) | 0.002 |
| Model 5 | 1 (Ref) | 0.348(0.128,0.568) | 0.386(0.109,0.613) | 0.581(0.358,0.804) | 0.003 |
| Model 6 | 1 (Ref) | -0.109(-0.468,0.249) | 0.059(-0.315,0.432) | -0.016(-0.386,0.355) | 0.977 |

Model 1: adjust cluster; Model 2: adjust age BMI, smoking status, marital status, work status, residency, ethnicity, household income level, Alcohol consumption, physical activity, sleep duration; Model 3: adjust use of fasting blood glucose, HbA1 serum insulin, HDL, LDL, triglycerides, CRP, Serum uric acid; Model 4: adjust blood pressure, general obesity; Model 5: adjust, family history of hypertension, family history of diabetes, use of antihypertensive medication, use of antidiabetic medication; Model 6: adjust total caloric intake.

**Table 3.** Multivariable Logistic Regression Analysis of the Association Between Dietary Patterns and Type 2 diabetes mellitus

|  | Cluster A  (High-fiber nutrient-dense) | Cluster B  (Staple–protein) | Cluster C  (Seafood–eggs) | Cluster D  (Sugary and processed foods) | P-value |
| --- | --- | --- | --- | --- | --- |
| Model 1 | 1 (Ref) | 1.312 (1.051,1.643) | 1.286(1.023,1.612) | 1.548(1.203,1.976) | 0.012 |
| Model 2 | 1 (Ref) | 1.343(1.082,1.661) | 1.256(1.018,1.559) | 1.492(1.164,1.915) | 0.009 |
| Model 3 | 1 (Ref) | 1.366(1.093,1.702) | 1.275(1.026,1.593) | 1.455(1.113,1.872) | 0.007 |
| Model 4 | 1 (Ref) | 1.386(1.112,1.738) | 1.307(1.058,1.634) | 1.425(1.116,1.834) | 0.005 |
| Model 5 | 1 (Ref) | 1.403(1.112,1.756) | 1.326(1.064,1.657) | 1.394(1.096,1.784) | 0.004 |
| Model 6 | 1(Ref) | 1.125(0.887,1.435) | 1.094(0.852,1.402) | 1.182(0.912,1.526) | 0.321 |

Model 1: adjust cluster; Model 2: adjust age BMI, smoking status, marital status, work status, residency, ethnicity, household income level, alcohol consumption, physical activity, sleep duration; Model 3: adjust use of fasting blood glucose, HbA1 serum insulin, HDL, LDL, triglycerides, CRP, serum uric acid; Model 4: adjust blood pressure, DM; Model 5: adjust, family history of hypertension, family history of diabetes, use of antihypertensive medication, use of antidiabetic medication; Model 6: adjust total caloric intake.

**Table 4.** Table 6. Stratified Logistic Regression of Obesity and Type 2 Diabetes Mellitus in Normal-Weight Participants

|  | | **DM** | | | **P** |
| --- | --- | --- | --- | --- | --- |
| Model 1 | 1 (Ref) | | -0.350 (-0.783,0.079) | 0.110 | |
| Model 2 | 1 (Ref) | | -0.385(-0.837,0.063) | 0.923 | |
| Model 3 | 1 (Ref) | | -0.417(-0.878,0.038) | 0.073 | |
| Model 4 | 1 (Ref) | | -0.442(-0.906,0.017) | 0.059 | |
| Model 5 | 1 (Ref) | | -0.504(-0.982,0.034) | 0.054 | |

Model 1: adjust obesity; Model 2:adjust age, smoking status, marital status, work status, residency, ethnicity, household income level, Alcohol consumption, physical activity, sleep duration; Model 3: adjust use of fasting blood glucose, HbA1, serum insulin, HDL, LDL, triglycerides, CRP, serum uric acid; Model 4: adjust cluster, general obesity; Model 5: adjust, family history of hypertension, family history of diabetes, use of antihypertensive medication, use of antidiabetic medication.

**Table 5.** Stratified Logistic Regression of Obesity and Type 2 Diabetes Mellitus in Overweight Participants

|  | | **DM** | | | **P** |
| --- | --- | --- | --- | --- | --- |
| Model 1 | 1 (Ref) | | -0.418 (-0.951,0.111) | 0.121 | |
| Model 2 | 1 (Ref) | | -0.508(-1.083,0.058) | 0.079 | |
| Model 3 | 1 (Ref) | | -0.517(-1.114,0.069) | 0.084 | |
| Model 4 | 1 (Ref) | | -0.516(-1.122,0.078) | 0.089 | |
| Model 5 | 1 (Ref) | | -0.544(-1.156,0.057) | 0.076 | |

Model 1: adjust obesity; Model 2:adjust age, smoking status, marital status, work status, residency, ethnicity, household income level, Alcohol consumption, physical activity, sleep duration; Model 3: adjust use of fasting blood glucose, HbA1, serum insulin, HDL, LDL, triglycerides, CRP, serum uric acid; Model 4: adjust cluster, general obesity; Model 5: adjust, family history of hypertension, family history of diabetes, use of antihypertensive medication, use of antidiabetic medication.

**Table 6.** Stratified Logistic Regression of Obesity and Type 2 Diabetes Mellitus in Obese Participants

|  | | **DM** | | | **P** |
| --- | --- | --- | --- | --- | --- |
| Model 1 | 1 (Ref) | | 0.088 (-0.294,0.469) | 0.652 | |
| Model 2 | 1 (Ref) | | 0.092(-0.294,0.478) | 0.642 | |
| Model 3 | 1 (Ref) | | 0.029(-0.370,0.428) | 0.887 | |
| Model 4 | 1 (Ref) | | 0.029(-0.372,0.431) | 0.884 | |
| Model 5 | 1 (Ref) | | 0.041(-0.364,0.444) | 0.844 | |

Model 1: adjust obesity; Model 2:adjust age, smoking status, marital status, work status, residency, ethnicity, household income level, Alcohol consumption, physical activity, sleep duration; Model 3: adjust use of fasting blood glucose, HbA1, serum insulin, HDL, LDL, triglycerides, CRP, serum uric acid; Model 4: adjust cluster, general obesity; Model 5: adjust, family history of hypertension, family history of diabetes, use of antihypertensive medication, use of antidiabetic medication.


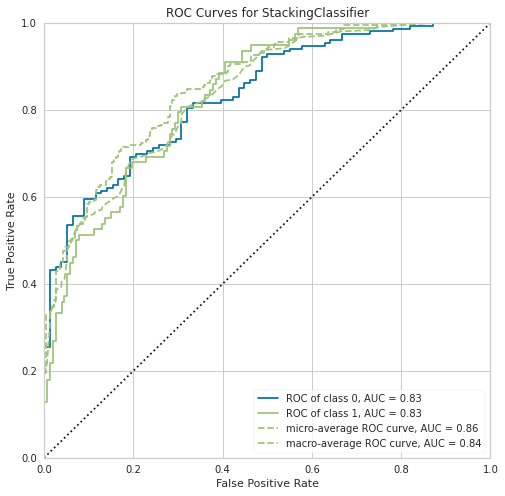


Supplementary Figure 1.ROC-AUC was used to evaluate the performance of the prediction model, both overall and for each T2DM-related feature. The model performance was calculated on the basis of the prediction on the randomly sampled test set.


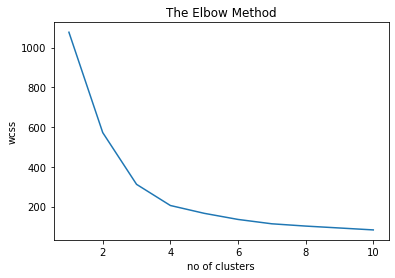


Supplementary Figure 2. Clustering reliability by elbow.

The horizontal axis represents the number of clusters, and the vertical axis represents the within-cluster sum of squares (WCSS) centroid, which is the sum of the squared distances between each data point and its assigned cluster centroid. The optimal number of clusters determined by the Elbow method is 3 or 4. Cluster number 4 was chosen considering data point distribution in the scatter plot.
